# Neuropathological Sequelae of Developmental Exposure to Antiepileptic and Anesthetic Drugs

**DOI:** 10.3389/fneur.2012.00120

**Published:** 2012-08-01

**Authors:** Christopher Andreas Turski, Chrysanthy Ikonomidou

**Affiliations:** ^1^2nd Faculty of Medicine, Charles UniversityPrague, Czech Republic; ^2^Developmental Brain Injury Laboratory, Department of Neurology, University of Wisconsin MadisonMadison, WI, USA

**Keywords:** glutamate, *N*-methyl-d-aspartate, GABA, apoptosis, neurogenesis, synaptogenesis, neurodevelopmental disorder

## Abstract

Glutamate (Glu) and γ-aminobutyric acid (GABA) are major neurotransmitters in the mammalian brain which regulate brain development at molecular, cellular, and systems level. Sedative, anesthetic, and antiepileptic drugs (AEDs) interact with glutamate and GABA receptors to produce their desired effects. The question is posed whether such interference with glutamatergic and GABAergic neurotransmission may exert undesired, and perhaps even detrimental effects on human brain development. Preclinical research in rodents and non-human primates has provided extensive evidence that sedative, anesthetic, and AEDs can trigger suicide of neurons and oligodendroglia, suppress neurogenesis, and inhibit normal synapse development and sculpting. Behavioral correlates in rodents and non-human primates consist of long-lasting cognitive impairment. Retrospective clinical studies in humans exposed to anesthetics or AEDs *in utero*, during infancy or early childhood have delivered conflicting but concerning results in terms of a correlation between drug exposure and impaired neurodevelopmental outcomes. Prospective studies are currently ongoing. This review provides a short overview of the current state of knowledge on this topic.

## Introduction

Short- and long-term deleterious effects, resulting from any interference with normal brain development, differ depending on the nature of interference, and the timing of the insult (Rodier, 1980). Despite variations in the rates of brain growth among mammals, comparisons of brain development between species are possible (Passingham, 1985; Bayer et al., 1993). Developmental ages are comparable when anatomical features and histological landmarks are similar in appearance in two species despite differences in chronological ages (Bayer et al., 1993). In the central nervous system, structures are built by cell proliferation, migration, and a sequence of steps called differentiation. Normal function requires a specific number of cells with the proper characteristics in the correct location (Rodier, 1980). Cell migration enables that neurons reach their final location where they establish physical contact between themselves and construct complex circuits (Rodier, 1994). Connections occur via the process of synaptogenesis. The developmental period of synaptogenesis is critical for the formation of the basic circuitry of the nervous system, although neurons are able to form new synapses throughout life (Rodier, 1995). Neurotransmitters modulate proliferation of neural stem cells, neuroblasts, and glioblasts, regulate migration and induce differentiation (Emerit et al., 1992; Retz et al., 1996; Levitt et al., 1997; Nguyen et al., 2001).

Neurogenesis produces about twice as many neurons in a given structure as the number of neurons that survive into adulthood. Excessive cells are eliminated by apoptosis or programmed cell death (Johnson and Deckwerth, 1993). Apoptosis is regulated by growth factors and cytokines, as well as by neurotransmitters and is executed by a number of intracellular proteins (Henderson, 1996; Kroemer et al., 2009). Any compound that interferes with these processes may trigger apoptotic degeneration of neurons that would not have otherwise been deleted from the developing brain, or may promote survival of unnecessary cells (Webb et al., 2001). Pruning, defined as a loss of synapses, also occurs physiologically in the developing brain. Such trimming of connections occurs late in childhood and adolescence (Webb et al., 2001).

## *N*-Methyl-d-Aspartate (NMDA) Antagonists and GABA-Agonists are Neurotoxins for the Developing Brain

A decade ago, it was reported that antagonists of the NMDA subtype of glutamate receptors cause widespread apoptotic neurodegeneration in the developing rat brain (Ikonomidou et al., 1999). NMDA receptors undergo a period of hypersensitivity through increased expression of specific receptor subunits (McDonald et al., 1988; Ikonomidou et al., 1989; Miyamoto et al., 2001). During this period, which extends from late fetal life to the first 2 weeks after birth in the rat, blockade of NMDA receptors with dizocilpine [(+)MK801], phencyclidine, ketamine, or 3-((6)-2-carboxypiperazin-4-yl)propyl-1-phosphonate (CPP) for a period of hours triggers widespread apoptotic neurodegeneration in the brain (Ikonomidou et al., 1999) and may lead to detrimental long-term effects (Behar et al., 1999; Popke et al., 2001). Treatment of infant rats with intraperitoneal injections of (+)MK801 resulted in a marked increase of degenerating neurons from threefold in the hypothalamus to 39-fold in the laterodorsal thalamus, when examined at 24 h by appropriate staining techniques (Ikonomidou et al., 1999). The form of cell death was found to be ultrastructurally identical with apoptotic cell death (Ikonomidou et al., 1999). Different brain regions display different age-dependent vulnerability profiles to NMDA receptor blockade, leading to distinct patterns of neuronal loss, depending on the timing of exposure. MK801 was shown to increase apoptosis either *in vivo* (Hsu et al., 2000), in motor neurons of a chick embryo preparation (Hsu et al., 2000), or in cultured neurons (Terro et al., 2000).

Further preclinical research revealed that a robust apoptotic response can also be triggered by agents (benzodiazepines and barbiturates) that mimic or potentiate the action of GABA at GABA_A_ receptors (Ikonomidou et al., 2000). Benzodiazepines and barbiturates, in a dose-dependent manner, triggered widespread cell death in the infant rat brain, which by ultrastructural analysis was apoptotic. The pattern of degeneration was similar for each GABAergic agent, but this pattern differed in several major respects from that induced by NMDA antagonists (Ikonomidou et al., 2000).

Evidence that ethanol, the most widely abused drug in the world, has NMDA antagonist and GABA_A_-agonist properties (Hoffman et al., 1989; White et al., 1990; Lovinger and White, 1991; Chandler et al., 1999) prompted evaluation of its ability to mimic the proapoptotic effects of other NMDA antagonists and GABA_A_ agonists. Transplacental ethanol exposure of the human fetus *in utero* can cause craniofacial anomalies, microcephaly, mental retardation, and neurobehavioral disturbances (Jones and Smith, 1973, 1975; Sulik et al., 1981; Stratton et al., 1996; Faingold et al., 1998). The fetotoxic effects of ethanol can also manifest as a partial syndrome comprised largely of neurobehavioral disturbances, without craniofacial malformations, referred to as fetal alcohol effects (FAE) or alcohol related neurodevelopmental disorder (ARND; Barr and Streissguth, 2001). During the brain growth spurt period, brief exposure to ethanol can trigger widespread apoptosis in the *in vivo* mammalian brain (Ikonomidou et al., 1999, 2000; Dikranian et al., 2001). While other mechanisms may play a role, the ability of ethanol to induce widespread apoptotic neurodegeneration throughout the forebrain, provides a more likely explanation than has been available previously for the reduced brain mass and lifelong neurobehavioral disturbances associated with the human fetal alcohol spectrum disorders (FASD; Ikonomidou et al., 1999, 2000; Dikranian et al., 2001; Olney et al., 2002a,b).

## Sedative, Anesthetic, and Antiepileptic Drugs Cause Neuronal Apoptosis in the Developing Brain

Sedative, anesthetic, and antiepileptic drugs (AEDs), routinely used in obstetric and pediatric medicine, trigger widespread apoptotic neurodegeneration throughout the developing brain when administered to immature rodents during the brain growth spurt (Mennerick et al., 1998; Ikonomidou et al., 2001; Jevtovic-Todorovic et al., 2003; Olney et al., 2004; Wang et al., 2006). Since the comparable period in humans extends from the sixth month of pregnancy to several years after birth (Dobbing and Sands, 1979, 1993), the hypothesis is formulated that there is a period in pre- and postnatal human development that lasts for several years, during which immature human neurons might be prone to commit suicide if exposed to sedative, anesthetic, or AEDs.

Neurotoxic effects of AEDs have been systematically studied in infant rodents (Bittigau et al., 2002; Glier et al., 2004; Manthey et al., 2005; Katz et al., 2007; Kim et al., 2007; Ikonomidou and Turski, 2010; Forcelli et al., 2011). The majority of them may cause apoptotic neurodegeneration in the developing rat brain at doses and plasma concentrations relevant for anticonvulsant treatment in humans. Apoptogenic effects were described for phenytoin, phenobarbital, diazepam, clonazepam, valproate, and vigabatrin (Bittigau et al., 2002). It was also determined that the apoptotic response to AEDs differs as a function of developmental age (Bittigau et al., 2002). Interestingly, topiramate elicited a neurotoxic effect in infant rat brain beginning at a dose of 50 mg/kg, which is above the reported effective anticonvulsant doses in infant rodent seizure models (Glier et al., 2004), suggesting a rather beneficial profile. Levetiracetam showed no neurotoxicity in the infant rat brain at all doses tested (Manthey et al., 2005).

## Mechanisms Implicated in Neurotoxicity of Sedative, Anesthetic, and Antiepileptic Drugs

Changes in synthesis of neurotrophins, i.e., brain derived neurotrophic factor, neurotrophins three and four as well as reduced levels of the active phosphorylated forms of extracellular signal regulated kinase (ERK1/2) and protein kinase B (AKT), were shown to partly underlie neurotoxicity (Bittigau et al., 2002; Hansen et al., 2004). Interestingly, 17β-estradiol counteracted inactivation of the ERK1/2 and AKT pathways and protected against neurotoxicity of some AEDs (Bittigau et al., 2002; Asimiadou et al., 2005).

The cell death process is Bax-dependent (Young et al., 2003) and involves decreased expression of phosphorylated extracellular signal regulated protein kinase (pERK; Young et al., 2008; Straiko et al., 2009; Sanders et al., 2010), and down regulation of Bclx_L_ (Yon et al., 2005; Sanders et al., 2010), mitochondrial injury and extra-mitochondrial leakage of cytochrome *c* (Young et al., 2003; Yon et al., 2005). This is followed by a sequence of changes culminating in activation of caspase-3 (Olney et al., 2002a,b). Studies using caspase-3 knockout mice suggest that commitment to cell death occurs before the caspase-3 activation step (Young et al., 2005), and this has led to the use of activated caspase-3 (AC3) immunohistochemistry as a standard means of mapping and quantifying neurons that undergo apoptotic neurodegeneration following exposure to apoptogenic drugs.

Involvement of endocannabinoids in the neurotoxic action of compounds interfering with NMDA receptors or GABA_A_ receptors has been demonstrated. Administration of Δ^9^-tetrahydrocannabinol, the principal psychoactive cannabinoid of marijuana, markedly enhanced proapoptotic properties of phenobarbital and MK801 in infant rats. Infant CB_1_ receptor knockout mice were less susceptible to the neurotoxic effect of ethanol. Furthermore, the CB_1_ receptor antagonist SR141716A ameliorated neurotoxicity of ethanol (Hansen et al., 2008).

## Neuroprotection with Lithium

It has been observed that alcohol, anesthetic drugs, and AEDs have an important property in common – all drugs in each category that have been tested, rapidly suppress phosphorylation of extracellular signal regulated kinases (ERK 1 and 2), which are known to regulate cell survival in the developing brain. This is well in advance of caspase-3 activation, which begins to become detectable at 2–3 h after apoptogenic drug administration (Olney et al., 2002a,b). In the developing mouse brain, lithium significantly increased ERK phosphorylation, and EtOH’s suppressant action on pERK was totally abolished when lithium was administered together with EtOH. The same effect was seen when lithium was administered together with anesthetic drugs (Straiko et al., 2009). Lithium also powerfully protected against the neurotoxic action of alcohol (Young et al., 2008) or anesthetic drugs, including propofol and ketamine (Straiko et al., 2009).

## Neurogenesis

Neurogenesis in the central nervous system occurs mainly prenataly in mammals, but continues to take place throughout life in restricted regions. Areas where substantial neurogenesis is found during the early postnatal period in rats and mice include the subventricular zone of the lateral ventricles, the cerebellum, the olfactory bulbs, and the subgranular zone of the hippocampal dentate gyrus (Cameron and McKay, 1999; Götz and Huttner, 2005). Enhanced neurogenesis has been associated with improved spatial memory performance in rodents (Kempermann et al., 1997, 1998).

Interference with neurotransmitters has been shown to influence neurogenesis in adulthood (Ge et al., 2006; Nacher and McEwan, 2006). However, knowledge on the influence of sedative and anticonvulsant drugs on early postnatal neurogenesis is limited.

In a recent study, the question of whether or not drugs that induce neuroapoptosis in the developing rodent brain also impair neurogenesis was addressed (Stefovska et al., 2008). The NMDA antagonist MK801, and the GABA_A_ agonists phenobarbital and diazepam were administered to infant rats; cell proliferation and neurogenesis were studied in the brain. Neurogenesis was quantified in the dentate gyrus on postnatal day 15, following treatment with MK801 or with phenobarbital on postnatal days 6–10. MK801, phenobarbital, and diazepam reduced numbers of newly born cells in the brain. In the dentate gyrus, many of the newly formed cells differentiated toward a neuronal phenotype. Phenobarbital and MK801 significantly reduced numbers of new neurons in that structure. At the age of 6 months, phenobarbital-treated rats had fewer neurons in the dentate gyrus and performed worse than saline-treated littermates in water maze learning and memory task. These findings show that blockade of NMDA receptor-mediated excitation, as well as enhancement of GABA_A_-receptor activation, impair cell proliferation, and inhibit neurogenesis in the immature rat brain (Stefovska et al., 2008). Comparable results have been reported by other groups (Chen et al., 2009; Shi et al., 2010; Fan et al., 2012).

## Anesthesia-Induced Impairment of Synapse Development and Remodeling and Cytoskeleton Formation

The detrimental effect of general anesthetics on synaptogenesis is age-dependent and most severe if the exposure occurs at the peak of synaptogenesis (Yon et al., 2005). General anesthetics administered to rats at postnatal day 7 cause severe, long-lasting functional and ultrastructural abnormalities of developing synapses in the hippocampus (Lunardi et al., 2010). No changes in synaptic lengths, the thickness of postsynaptic densities, or the number of presynaptic vesicles have been found, but significant decreases in synapse volumetric densities, paucity of multiple synaptic boutons, and long-lasting decreases in the strength of inhibitory synaptic neurotransmission were reported (Lunardi et al., 2010, 2011).

In a recent study it was shown that exposure of mice to general anesthesia at the peak of synaptogenesis causes significant reductions in dendritic filopodial spines and synapse formation in hippocampal neurons (Head et al., 2009). Briner et al. (2010) have found that exposure of mice to general anesthesia on postnatal day 16 results in a significant increase in the density of dendritic spines in the basal and apical dendrites in pyramidal neurons of the prefrontal cortex. These results indicate strong age-dependent vulnerability of synaptic spines and boutons during early stages of brain development.

The actin cytoskeleton determines neuronal and glial morphology and function. Lunardi et al. (2011) and others (Lemkuil et al., 2011) explored whether general anesthesia modulates those signaling pathways. Immature primary glia in culture were exposed to an inhalational anesthetic, isoflurane. This led to profound disruption of actin cytoskeleton organization, triggered by significant disruption of the Ras homolog gene family member A (RhoA)–myosin light chain (MLC) signaling pathway (Lunardi et al., 2011). RhoA is the most influential member of a family of small GTPases (Bishop and Hall, 2000). RhoA functions as a molecular switch, cycling between an inactive GDP-bound state and an active GTP-bound state, and regulates the phosphorylation of MLC proteins (MLC-P), which, in turn, promote organization of the actin cytoskeleton in actin stress fibers (ASFs), and the formation of focal adhesions. It appears that isoflurane causes severe disruption of actin cytoskeletal sculpting by modulating the RhoA signaling pathway, that is, by downregulating activated RhoA and MLC-P. This ultimately leads to impairment of astrocyte morphological differentiation and maturation (Lunardi et al., 2011). Similar disruption of the actin cytoskeleton was observed when primary glia were exposed to alcohol (Guasch et al., 2003; Minambres et al., 2006).

Immature neurons appear to be vulnerable to isoflurane-induced disruption of the actin cytoskeleton as well. Lemkuil et al. (2011) have shown that relatively brief exposure to isoflurane (up to 120 min) resulted in significant cytoskeletal depolymerization and disorganization. They report that actin depolymerization is brain derived neurotrophic factor/p75 neurotrophin receptor-mediated, and relies on upregulation of activated RhoA, as shown in immature astroglia. This suggests that the isoflurane-induced impairment of actin cytoskeletal sculpting observed in both immature neurons and glial cells is initiated by two apparently opposite effects on the activated RhoA protein. The result is impairment of proper neurite growth (Lemkuil et al., 2011) and glial process formation (Lunardi et al., 2011), necessary for neuron–neuron and neuron–glia interactions.

## Anesthesia-Induced Impairment of Glial Maturation and Growth

Given that neuronal well-being and development depend on the integrity and proper functioning of astroglia (Ullian et al., 1991; Barres, 2008), the most abundant group of glial cells, it is important to understand whether and how anesthesia may disturb astroglia development. Exposure of cultured immature astroglia to isoflurane causes significant impairment of their growth and postpone timely astroglial morphological transformation. Isoflurane causes significant disturbances in ASF formation and the content of paxillin, a focal adhesion protein that is linked to ASFs and enables anchoring to the extracellular matrix, and cytoskeletal organization (Lunardi et al., 2011). This hinders the formation of an organized glial network.

Apoptotic cell death is not a significant component of isoflurane toxicity in astroglia (Lunardi et al., 2011). However, recently Brambrink et al. (2012b) reported that isoflurane causes significant apoptotic changes in developing oligodendroglia *in vivo* in the non-human primate brain. Thus, it is reasonable to propose that general anesthesia is most likely detrimental to glial development also, and that it exerts its detrimental effects via different mechanisms, that are unique to each subpopulation of glial cells.

## Evidence for Adverse Effects of Anesthetics in Humans and Non-Human Primates

Studies conducted by the National Center for Toxicology Research (NCTR) of the Food and Drug Administration (FDA) have demonstrated that exposure to ketamine – the prototypical NMDA receptor antagonist – resulted in increased neuronal cell death in non-human primates. Doses of ketamine which produced a light surgical plane of anesthesia for either 9 or 24 h resulted in neuroapoptosis in 5-day-old rhesus monkeys. Neuroapoptosis in the brain of the fetus was also evident when pregnant rhesus monkeys were exposed to ketamine for 24 h on day 122 of gestation (equivalent to the third trimester of human pregnancy), but no neuroapoptosis was noted following administration of ketamine on postnatal day 35 (Slikker et al., 2007). Neuroapoptosis has also been demonstrated in primates who were given isoflurane (predominantly a GABAergic agent) on postnatal day 6 (Brambrink et al., 2012a). The FDA and others are currently conducting studies in animals to address the neurocognitive and neurobehavioral effects of anesthetic-induced apoptosis.

An operant test battery is used to evaluate the cognitive function of rhesus monkeys exposed to a dose of ketamine sufficient to produce a light surgical plane of anesthesia for 24 h on postnatal day 5 or 6. Compared with controls, ketamine-treated animals have lower training scores for at least 10 months after the administration of ketamine (Paule et al., 2011).

It is not known how the data from rodents or primates translate to humans, but such findings raise questions that require further studies.

A retrospective cohort analysis followed a birth cohort of 383 human children who underwent inguinal hernia repair during the first 3 years of life, and compared them with 5050 children in a control sample who had undergone no hernia repair before the age of three (DiMaggio et al., 2009). The children who underwent hernia repair were twice as likely to be given a diagnosis of a developmental or behavioral disorder. A population-based, retrospective, birth cohort study examined the educational and medical records of children who were exposed to a single anesthetic (*n* = 449), two anesthetics (*n* = 100), or more (*n* = 44). No increased risk of learning disabilities was found with a single anesthetic but, an increasing risk of learning disabilities was associated with two or more anesthetics. The risk of learning disabilities also increased with greater cumulative exposure to anesthesia (Wilder et al., 2009).

At present, there is not enough information to draw any firm conclusions regarding an association between anesthetic exposure and subsequent learning disabilities, and additional studies are warranted.

## Clinical Evidence for Adverse Outcomes Following Prenatal or Early Childhood Exposure to AEDs in Humans

Antiepileptic drugs are among the most common causes of fetal malformations. These include neural tube defects, orofacial clefts and digital anomalies, growth retardation, developmental delay, and microcephaly (Speidel and Meadow, 1972; Strickler et al., 1985; Jones et al., 1989; Buehler et al., 1990; Zahn, 1998; Holmes et al., 2001).

It has also been shown that AEDs may have adverse effects on human intellect when given to treat seizures in pregnant women, infants, and toddlers. Long-lasting neurobehavioral effects in humans following *in utero* exposure to phenobarbital, such as impaired cognitive development and lower IQ scores (Reinisch et al., 1995), have been reported. Valproic acid is a clear animal and human teratogen (Winter et al., 1997; Holmes et al., 2001; Wyszynski et al., 2005). The nervous system appears to be very sensitive to the developmental toxicity of valproic acid. Neural tube defects, specifically spina bifida, occur at a high rate upon *in utero* exposure to this compound (Lindhout and Schmidt, 1986). Carbamazepine has been found to be teratogenic in humans, and the pattern of malformations (facial dysmorphic features, microcephaly, and growth retardation) resembles that of other anticonvulsants (Jones et al., 1989; Matalon et al., 2002). The fetal hydantoin syndrome in humans is characterized by facial dysmorphologies, growth retardation and other anomalies, and similar effects have also been seen in rodents (Finnell and Chernoff, 1984; Finnell et al., 2002). Microcephaly, learning disabilities, and decreased IQ scores have also been reported in humans (Adams et al., 1990; van Overloop et al., 1992; Dessens et al., 2000).

The majority of studies investigating the effects of prenatal AED exposure were performed in children below age five. In many studies, a trend toward lower developmental scores was reported, although in a few studies no adverse effects could be shown (Rovet et al., 1995). In a longitudinal study, children exposed to carbamazepine and phenytoin had lower developmental and language scores, compared to controls. In children receiving carbamazepine, a deficit in language development was not evident until an age beyond 3 years, suggesting that specific cognitive deficits may first become apparent when the child is older (Adab et al., 2001).

Inconsistent results have also been obtained in studies that have investigated the long-term effects of AED exposure in children older than 5 years (Hanson et al., 1976; Gaily et al., 1988; Dessens et al., 2000; Mawer et al., 2002). Some studies have reported specific cognitive deficits in visuo-spatial functioning (Hanson et al., 1976; van der Pol et al., 1991), spelling and linguistic abilities (Leavitt et al., 1982; Scolnick et al., 1994; Adab et al., 2001).

Intrauterine exposure to phenytoin, phenobarbital, polytherapy, valproate, and carbamazepine is associated with lower intellectual functioning (Leavitt et al., 1982; Lösche et al., 1994; Scolnick et al., 1994; Vinten et al., 2005). Carbamazepine appears to be the least developmentally neurotoxic compound among the major AEDs. Although in one study, mild mental retardation has been reported in children exposed *in utero* to carbamazepine (Ornoy and Cohen, 1996), no neurologic, or IQ differences were found by other investigators (van der Pol et al., 1991; Scolnick et al., 1994).

In humans, *in utero* exposure to valproic acid has been associated with developmental delay, mental retardation, cognitive impairment, and other behavioral deficits (Sulzbacher et al., 1999; Thorp et al., 1999). In a retrospective study exploring neuropsychological effects of exposure to anticonvulsant medication *in utero*, children exposed *in utero* to valproate had a significantly lower IQ when compare to children exposed to other AEDs, or not exposed at all. The same children were more likely to have an IQ below 69 and more likely to have memory impairment when compared to other groups (Vinten et al., 2005).

In addition, postnatal exposure to AEDs during the first years of life may be harmful for cognitive development. In several studies it was shown that therapy with barbiturates during the first 3 years of life may cause cognitive impairment that persists into adulthood (Diaz et al., 1977; Yanai et al., 1989; Farwell et al., 1990; Reinisch et al., 1995).

Meador et al. (2009) conducted a prospective clinical study of the impact of *in utero* exposure to AED on cognitive abilities. At 3 years of age, children who had been exposed to valproate *in utero* had significantly lower IQ scores than those who had been exposed to other AEDs. After adjustment for maternal IQ, maternal age, AED dose, gestational age at birth, and maternal preconception use of folate, the mean IQ was 101 for children exposed to lamotrigine, 99 for those exposed to phenytoin, 98 for those exposed to carbamazepine, and 92 for those exposed to valproate. On average, children exposed to valproate had an IQ score nine points lower than the score of those exposed to lamotrigine, seven points lower than the score of those exposed to phenytoin, and six points lower than the score of those exposed to carbamazepine. They concluded that *in utero* exposure to valproate, as compared with other commonly used AEDs, is associated with an increased risk of impaired cognitive function at 3 years of age. This finding supports a recommendation that valproate not be used as a first-choice drug in women of childbearing potential.

Morphological changes in the brains of subjects exposed *in utero* to AEDs have also been analyzed. A group of healthy young adults with prenatal AED exposure (PAE) and a group of age-matched unexposed healthy controls were subjected to magnetic resonance imaging (MRI) of the brain and structural differences between the two groups were studied by means of voxel based morphometry (Ikonomidou et al., 2007).

Regional decreases of gray matter volumes were found in PAE subjects in the area of the lentiform nucleus, including both pallidum and putamen bilaterally, and the hypothalamus. Thus, prenatal exposure to AEDs causes subtle morphological changes in gray matter of the human brain, consisting of lower gray matter volumes in the basal ganglia and the hypothalamus.

## Conclusion

The experimental data reviewed provide compelling evidence that sedative, anesthetic, and AEDs may alter normal brain development by influencing cell proliferation, neurogenesis, migration, programmed cell death, synaptogenesis, synaptic plasticity, and possibly myelination in the developing brain. A critical period of high vulnerability of the brain to AEDs is the brain growth spurt period.

These findings raise concerns regarding current clinical practice of employing these drugs to pregnant women, infants, and children. They call for the design of novel sedative, anesthetic, and AEDs, adjunctive neuroprotective therapies, and generation of new clinical data by means of well designed clinical trials to guide clinical practice. The findings that β-estradiol and lithium ameliorated apoptosis in experimental animal models is encouraging in that respect, and may lead to new therapeutic avenues that will allow to prevent neurotoxicity of sedatives and anesthetics.

Of note is also that experimental studies demonstrated favorable profile for carbamazepine and lamotrigine. Topiramate is an AED with a favorable therapeutic index in terms of the separation between anticonvulsant activity and neurotoxic/proapoptotic effects in the developing rat brain (Glier et al., 2004). Even more encouraging was the finding that levetiracetam does not demonstrate a proapoptotic effect (Manthey et al., 2005). Thus, there is hope that it will be possible to design age-specific antiepileptic therapies and at the same time avoid or minimize neurotoxic side effects in the vulnerable age groups.

## Conflict of Interest Statement

The authors declare that the research was conducted in the absence of any commercial or financial relationships that could be construed as a potential conflict of interest.
